# Bacteriological quality and safety of bottle food and associated factors among bottle-fed babies attending pediatric outpatient clinics of Government Health Institutions in Arba Minch, southern Ethiopia

**DOI:** 10.1186/s41043-023-00387-1

**Published:** 2023-05-26

**Authors:** Alebachew Marege, Belayneh Regassa, Mohammed Seid, Dagimawie Tadesse, Munira Siraj, Aseer Manilal

**Affiliations:** Department of Medical Laboratory Science, College of Medicine and Health Sciences, Arba Minch University, Arba Minch, Ethiopia

**Keywords:** Bottle feeding, Supplementary food, Microbial load, Unsafe food, Bacterial contamination, Critical control point, Babies, Infant and young children, Arba Minch, Ethiopia

## Abstract

**Background:**

Microbial contamination of baby bottle food has been identified as a significant public health concern, especially in developing countries, but it remains overlooked. Therefore, this study aimed to determine microbiological hazards, compliance with hygiene practices, and critical control points of contamination in baby bottle food in Arba Minch, southern Ethiopia.

**Objective:**

To evaluate the bacteriological quality and prevalence of foodborne pathogens in baby bottle food and to identify associated factors among bottle-fed babies attending three government health institutions in Arba Minch, southern Ethiopia.

**Methods:**

A cross-sectional study was conducted between February 24 and March 30, 2022. A total of 220 food samples, comprising four types prepared with different sources of materials, were collected from systematically selected bottle-fed babies attending health facilities. The data on sociodemographic characteristics, food hygiene, and handling practices were solicited by face-to-face interview using a semi-structured questionnaire. Food samples (10 mL) were quantitatively analyzed for total viable counts (TVC) and total coliform count (TCC) and qualitatively for the presence of common foodborne bacterial pathogens. Data were analyzed using SPSS; ANOVA and multiple linear regression analyses were done to identify factors influencing microbial counts.

**Results:**

Results revealed that the means and standard deviations of TVC and TCC were 5.3 ± 2.3 log_10_ colony forming units (CFU)/mL and 4.1 ± 2.6 log_10_ CFU/mL, respectively. Of the various food samples analyzed, 57.3 and 60.5% had a TVC and TCC above the maximum acceptable limits, respectively. The result of the ANOVA showed that there was a significant difference in the mean score of TCV and TCC among the four types of food samples (*p* < 0.001). *Enterobacteriaceae* were found in the majority of positive food samples (79.13%), followed by Gram-positive cocci (20.8%). *Salmonella* spp., diarrheagenic *Escherichia coli,* and *Staphylococcus aureus* were the common foodborne pathogens detected in 8.6% of tested foods. The regression result revealed that the type of baby food, hand washing practices of mothers or caregivers, and sterilizing and disinfecting procedures of feeding bottles are independent determinants of bacterial contamination (*p* < 0.001).

**Conclusion:**

The high microbial load and the presence of potential foodborne bacterial pathogens in the bottle food samples analyzed indicate unsanitary practices and the potential risk of exposure to foodborne pathogens in bottle-fed babies. Thus, interventions such as educating parents about proper hygiene practices, sterilizing feeding bottles and limiting bottle feeding practices are critical to reducing the risk of foodborne to bottle-fed infants.

**Supplementary Information:**

The online version contains supplementary material available at 10.1186/s41043-023-00387-1.

## Introduction

Bottle feeding is the act of providing supplementary foods (liquid, soft, or semi-solid) through a feeding bottle with a nipple, regardless of whether the infant is breastfed or not [1–3]. Bottle feeding is practiced by 12 to 38% of caregivers or mothers in developing countries for medical or practical reasons, including insufficient breast milk, infants born to HIV-positive mothers, ease of feeding, and calming crying babies [1, 2, 4, 5]. However, the World Health Organization discourages bottle feeding due to concerns about the contamination of complementary foods and baby bottles [6–8].

Several studies indicate that unhygienic bottle feeding practices and improper preparation of complementary foods increase the risk of foodborne illness and mortality [3, 9, 10]. The microbial criteria were utilized as a risk management tool to assess the quality and estimate the food safety risk [11]. Albeit commercially manufactured powdered infant formula (PIF) is the primary focus of microbiological risk assessment in baby supplements, statistics indicate that homemade, traditional infant and children foods contain significant levels of microbial contamination [9, 12–14]. Most published literature mentions that the enumeration of indicator organisms such as aerobic mesophilic bacteria, coliform bacteria, and fecal coliform bacteria are the most common microbial criteria used for evaluating the microbial quality of baby supplementary foods [10, 14]. Furthermore, the detection and quantification of members of the *Enterobacteriaceae* have also been used to highlight the risk of exposure to opportunistic and foodborne pathogens in baby food [10, 14, 15]. Numerous studies were done in Ethiopia [16], Logos [13], Korea [17, 18], Nigeria [17], Gambia [14], Kenya [19], and Pakistan [7] identified unhygienically prepared supplementary foods using a single or combination of hygienic indicators, such as aerobic plate count (APC) (> 10^5^ CFU/mL) and TCC (> 10^2^ CFU/mL) [3, 10]. Similarly, contamination of food samples with organisms of fecal origin was reported in more than half of the tested samples, as evidenced by the presence and high load of coliforms or *E. coli* [14, 16, 19, 20]. It is reported that weaning foods are more than ten times more likely to be contaminated than the potable water used by children [17].

One of the gravest public health food safety concerns in bottle feeding practice is the risk of exposure to potential foodborne pathogens such as *Enterococcus* spp.,* S. aureus, Salmonella* spp., *Shigella* spp., and diarrheagenic *E. coli* [11, 15, 21]. Infants and babies are highly vulnerable to these pathogens due to their underdeveloped immune systems and lack of competing gut flora [10].

Microorganisms may contaminate food at any point along the farm-to-fork continuum. The contamination of supplementary foods in low and middle-income countries has been attributed to unhygienic processing and handling practices, the level of awareness among caregivers, socioeconomic status, the water source, and the equipment and utensils [9, 15]. However, the prevalence of baby food contaminated with indicator organisms and foodborne pathogens varies across geographies, study settings, and food types [19, 22]. Moreover, the microbial quality, the estimated food safety risk, and the identified factors that influence microbial growth and multiplication in PIF are inconclusive for planning preventive and control measures due to methodological disparities in the literature [11, 17]. Most information on this topic comes from studies conducted in the 1980s before the implementation of several food safety measures [14, 16]. Earlier research on child food hygiene in low-income countries such as Ethiopia had limited analyses of microbial quality and minimal data on factors that independently predict microbial contamination, making the effectiveness of home-based interventions to reduce microbial contamination still dubious [16, 22]. Contradictory evidence exists regarding the efficacy of cleansing, sterilization, and disinfection in reducing microbial contamination, which can paradoxically impact basic hygiene practices such as hand washing [23, 24]. More credible evidence has supported identifying critical control points for factors affecting microbial quality and opportunities to improve exposure risk and child health outcomes [25, 26]. In fact, a better understanding of the level of microbial contamination in baby bottle food and associated factors in high-risk populations would be crucial for planning and developing effective preventive interventions in the localized context [26].

To the best of our knowledge, this is the first study that provided evidence on the microbial quality and safety of supplementary baby bottle food and the factors associated with contamination in Arba Minch, southern Ethiopia. Therefore, the finding can fill the research gaps and highlight the food safety risk associated with bottle feeding practices and the required food safety hygiene. In light of the above-cited background, the main objective of this study was to evaluate the bacteriological quality of baby feeding bottle foods samples using hygiene indicator parameters total viable counts (TVC) and total coliform counts (TCC) and assessing the food safety by detecting the common foodborne pathogens in food samples at the time of consumption. As a secondary objective, the study also assessed a load of bacterial contamination in relation to participants' demographics and food hygiene practices involving mothers/caregivers and bottle-fed babies attending three health institutions in Arba Minch, southern Ethiopia.

## Material and methods

### Study setting, design and period

This institution-based cross-sectional study was undertaken from February 24 and March 30, 2022, involving the three health institutions found in Gamo zone, Arba Minch. The health institutions were Arba Minch General Hospital, Dilfana Primary Hospital, and Secha Health Centre and found about 5 km apart. Arba Minch town is located in the Gamo zone, 505 km from the capital city, Addis Ababa. Based on figures published by the Central Statistical Agency in 2007, the town has a total population of 74,843, of which 39,192 are males, and 35,651 are females [27].

### Source and study population

The source populations were all caregivers, or mother-and-baby pairs, attending the health institutions. However, the study populations were a combination of mothers, caregivers, and babies who were bottle-fed or had a feeding bottle with adequate amount of supplementary food. In addition, the study included mothers who were permanent residents (who had lived in the study area for at least six months) and who had infants, babies or young children under 24 months with a feeding bottle.

### Eligibility criteria

All caregivers, mothers, and children who carried feeding bottles into health institutions were eligible. Those who exhibited a willingness to participate and provided written consent were included. Hospitalized children and those with insufficient (< 10 mL) leftover bottle food for sampling were excluded.

### Sample size calculation and sampling techniques

The sample size was determined by OpenEpi software version 3.03 using formula for single population proportion. The proportion (P) of coliform (*E. coli*) contamination (*P* = 0.8) was chosen from a previous study conducted elsewhere [28]. After considering a 95% of confidence interval (*z* = 1.96) and 5% marginal error (*d* = 0.05), and a 10% non-response rate, the final sample size was consolidated to be 220.

Participants were proportionally allocated to each health institution by taking the number of patient flow in the previous year of similar month and dividing to the total sample size and multiplying by 100. Accordingly the number of participants included from Arba Minch General hospital were 110, from Dilfana primary hospital (*n* = 82) and Secha health center (*n* = 28). Systematic random sampling technique was applied to select each study participants, and the K^th^ value of 3 was used as sampling interval.

### Outcome variables and measurements

The primary outcome of this study was microbial quality (load) and safety of homemade bottle foods at the time of consumption among bottle-fed babies. The secondary outcome of interest was the analysis of microbial flora and identifying independent factors that influence the microbial load of baby foods. The microbial quality of the food samples was evaluated based on the evaluation of the food hygiene indicators/surrogate microorganisms. Of the various microbial parameters, this study included the TVC and TCC as indicators of microbial quality and level of hygiene in the process of food preparation, handling and storage. The bacteriological quality of each food sample is categorized as acceptable, marginally acceptable, and unacceptable as per the standard. The food safety measurement includes isolation and identification of foodborne pathogens such as *Salmonella* spp., *Shigella* spp., *S. aureus* and diarrheagenic *E. coli*, and results were dichotomized as a binary variable (positive and negative) [29, 30].

### Independent variables/covariates

Variables which are considered independent predictors in this study include sociodemographic characteristics of caregiver /maternal–child pairs, types of baby food and food hygiene knowledge and practices of caregivers/mothers on food safety.

### Data collection method and instruments

Data were collected using an interviewer-administered structured questionnaire (Additional file 1: Table S1 questionnaire). The questionnaire was adopted from earlier studies with slight alterations to fit the current study context [29, 31, 32]. The content of the questionnaire in part I included information on sociodemographic characteristics, access to electricity, toilets, refrigerators, water supplies, and other sanitary facilities, as well as the child's clinical status; in part II, the types of baby bottle food; in part III, the food safety knowledge of the caregivers or mothers; and in part IV, the food hygiene practices of the caregivers or mothers. A face-to-face interview was conducted with the mothers or caregivers in their native language. A copy of the English version of the questionnaire is provided in Additional file 1: Table S1.

### Data collection procedure

#### Microbiological evaluation of food hygiene indicators

##### Sample collection and transportation

The microbiological quality and safety of complementary food in the baby feeding bottle were determined by collecting approximately ten milliliters (10 mL) food samples from consented participants attending health institutions in southern Ethiopia. In brief, common complementary food samples (i.e., powdered infant formulary (PIF), cereal blend, cow milk, and fruit juice) were directly collected to dried, sterile, screw-capped universal tube labeled with code number at the time of enrollment. The samples were immediately placed in cooler box containing ice packs and transported to microbiology and parasitology laboratory, department of medical laboraoty sciences for microbiological processing within two hours as per the standard protocols described elsewhere [29, 30].

#### Sample preparation for analysis

For the quantitative analysis, the serial dilution of the sample suspension was done according to the procedure mentioned in ISO 6887-1: 2017 [29]. In brief dilutions were prepared by diluting ten milliliters of the food sample with 90 mL sterile saline peptone water and mixed up well to obtain 10^–1^ dilutions. From the primary dilution serial dilution was prepared up to 10^–5^ and plates were inoculated in triplicate by spread plate techniques. Then the plates were incubated at the temperature and times corresponding to each microbial indictors groups and the mean number of colonies counted was expressed as Colony Forming Units /milliliters (CFU/mL).

##### Enumeration of total viable count

The enumeration of TVC from the food sample was performed according to the previously reported method [30]. Briefly, 0.1 mL of each serially diluted homogenate was spread on the surface of nutrient agar plates (Oxoid) using separate sterile pipettes for each dilutions. Sterile spreader was used to spread the sample on the plates and plates were incubated 35 ± 2 °C aerobically for 24 h. Plates with 30–300 colonial count were considered appropriates and colony was counted using a colony counter (Astor 20 Colony Counter, Astori Tecnica, Italy). The arithmetic mean of final count has calculated the results using Eq. 1.

1 $$N=\frac{\sum C}{V [n1+(0.1n2)]d}$$

where ∑*C* = the sum of colonies counted on the plates, *n*1 = the number of dishes retained in the first dilution, *n*2 = the number of dishes retained in the second dilution, *d* = the value corresponding with the dilution from which the first counts were obtained (e.g., 10^–2^).

##### Enumeration of total coliform count

The TCC was determined by the spread plate method [3]. Briefly, 0.1 ml of each serially diluted homogenate was spread on the surface of MacConkey agar using separate sterile pipettes for each dilutions and plates were incubated at 35 ± 2 °C for 24 h. Plates containing colonies in the countable ranges (30–300) were considered, and mean results were expressed as CFU/mL. The results were interpreted according to standards set by the International Commission on Microbiological Specifications for Foods and the Center for food safety [30]. Finally, the quality of baby feeding bottle food was classified into acceptable, marginally acceptable, and unacceptable using the standard criteria indicated in the Additional file 2.

##### Microbial flora analysis

Microflora analyses were performed following the procedure described by Erku W-A, and Ashenafi M and Cheesbrough M [16, 33]. Briefly after the enumeration of TVC, about ten colonies were randomly picked from plates, separately inoculated into the nutrient broth (Oxoid), and incubated at 32 °C overnight. Cultures were identified to the genus level using Gram-staining and a battery of biochemical tests as described previously [16, 33].

##### Evaluation of food safety indicators

The potential foodborne pathogenic bacteria such as *E. coli*, *Salmonella* spp.*, Shigella* spp., and *S. aureus* were isolated and identified as per the methodology described elsewhere. In addition, the agglutination test was done to classify *E. coli* using antisera group O [30, 33].

### Data quality assurance

One day of training was given to data collectors on the data and sample collection procedures. In addition, a pre-test was conducted on 5% of the total sample size in Jinka General Hospital before the actual data collection for checking the validity of data collection tools. The quality was further assured by checking the completeness of the collected raw data (the laboratory and demographic data) before entry into the database. Furthermore, the quality and reliability of data generated by laboratory assay were assured following standard operating procedures throughout the sample collection and testing procedure. Moreover, culture media used for the isolation and enumeration of microorganisms was subjected to quality control tests using reference strains such as *S. aureus* (ATCC 25923), *E. coli* (ATCC 25922), and *P. aeruginosa* (ATCC 27853). Data reporting and final writing quality and completeness were assessed by using the STROBE checklist (Additional file 3: Table S2).

### Data management and analyses

Data were cleaned, checked, and analyzed using Statistical Package for the Social Sciences (SPSS) (IBM SPSS version 25) and Microsoft Excel statistical software 2016. The frequencies and descriptive statistics, such as mean, standard deviation, and minimum and maximum, were used to summarize the sociodemographic data of the caregiver-babies pair and the bottle food microbiological load, food hygiene, and safety practices of the caregiver. A descriptive analysis of data was conducted, and the difference between groups was assessed using chi-square or Fisher’s exact test or unpaired *t*-test or Mann–Whitney *U* test as appropriate.

A TVC and a TCC of bottle food were measured continuously based on the logarithmically transformed data. After the normal distribution was checked, all bacterial counts were transformed to a logarithmic scale, and the results were expressed as log_10_ CFU/mL. The mean log_10_ CFU/mL value was calculated on the assumption of a log-normal distribution, and an analysis of variance (ANOVA) test was used to analyze the statistical differences in bacterial counts according to sociodemographics and food hygiene and safety practices with statistically different means separated using Tukey's Honestly. The F-ratio computed from Welch’s statistics was used to resolve the mean difference, and the statistically significant difference was set at a 95% confidence interval (*p* < 0*.*05). Furthermore, a multiple linear regression test was used for the estimation of least square regression equation between dependent variables (total coliform count) and other independent variables and control confounding factors. Before linear regression analysis, a preliminary data check was done to ensure the assumption of no multicollinearity. In this study, multicollinearity was checked by using the Pearson correlation coefficient, the tolerance level and the variance inflation factors (VIF). The stepwise regression method was used to select the best predictors independent variables that would result in the best possible model fit. The *t*-statistic was used to test whether a particular variable contributes significantly to the regression model. The equation of regression lines across groups was tested. The multiple correlation coefficient, standard error of an estimated value, standardized and unstandardized regression coefficients, and significance of coefficient and *p*-value were reported. The regression equations were evaluated using the measure of variability, including adjusted R. Missing values were not inferred.

### Ethical consideration

Ethical approval was obtained from the Institutional Review Board of Arba Minch University, College of Medicine and Health Sciences (Ref. No. IRB/1204/2022). A letter of permission was also secured from the head of each health institution. Written Informed consent from each caregiver or mother and age-appropriate assent signed by the baby's guardian prior to performing any procedures specific to the study. The information obtained from each participant was kept confidential, any participant identifying information was coded, and all data were kept in a lockable box.

## Results

### Characteristics of study participants

Table 1 outlines the sociodemographic characteristics of caregivers/mothers and babies who participated in the study. A total of 212 pairs of caregivers/mothers and babies from the three health institutions participated, with a response rate of 100%. About 85% (*n* = 87) of primary child caregivers were females, while female babies accounted for 46.8% (*n* = 103). The mean ages of caregivers and babies were 30 ± 6.4 years and 30 ± 6.4 months, respectively. Nearly three-quarters of the mothers or caregivers (59.1%) were between the age of 20 and 30, and 45% (*n* = 99) of the babies were between the age of 0 to 5 months. More than 50% of caregivers or mothers were homemakers (53.6%). Most of the respondents were the biological mothers (76.4% 168/220). On the other hand, less than one-quarter of respondents completed their education up to the college level (20.9%). The monthly income of 25.5% of caregivers or mothers exceeded 5000 birrs. Invariably, all the participants had access to a toilet and electricity, and 90.9% had access to potable water from the tap; however, only 60% had access to a refrigerator. A greater number of the study participants, i.e., 50% (*n* = 110), were recruited from the Arba General Minch Hospital, while 37.3 and 12.7% were from Dilfana Primary Hospital and Secha Health Center, respectively. Regarding the various medical reasons for attending health institutions, 72 (32.7%) of them visited for routine vaccinations, 65 (29.5%) for gastrointestinal illness, 49 (22.3%) for respiratory illness, 27 (12.3%) for febrile illness, 3 (1.4%) for skin and soft tissue disease, and 4 (1.8%) for other medical complaints (Table 1).

**Table 1:** Socioeconomic characteristics of study participants attending health institutions in Arba Minch, southern Ethiopia, 2022 (*n* = 220)

Characteristics	Frequency (*n*)	Percent (%)
Age category of caregiver		
< 20	8	3.6
20–30	130	59.1
31–40	70	31.8
> 40	12	5.5
Sex of caregivers		
Male	33	15
Female	187	85
Occupation of caregivers		
Housewife	118	53.6
Merchant	27	12.3
Farmer	1	0.5
Labor work	16	7.3
Government employee	51	23.2
Student	7	3.2
Relation of caregivers to the child		
Mother	168	76.4
Father	30	13.6
Sibling	11	5
Homemaker	4	1.8
Grandparents	7	3.2
Sex of babies		
Male	117	53.2
Female	103	46.8
Age (per month) of babies		
0–5	78	35.5
6–8	54	24.5
9–24	88	40
Medical reasons to visit health institutions		
Vaccination	72	32.7
Gastrointestinal illness	65	29.5
Respiratory illness	49	22.3
Febrile illness	27	12.3
Skin and soft tissue disease	3	1.4
Others	4	1.8
Educational status		
Illiterate	56	25.5
Reading and writing only	28	12.7
Primary school	52	23.6
Secondary school	38	17.3
College/university	46	20.9
Estimated monthly income of the family		
500–1000	16	7.3
1001–2000	52	23.6
2001–3000	47	21.4
3001–4000	17	7.7
4001–5000	32	14.5
> 5000	56	25.5
Source of drinking water		
Ground well	6	2.7
Tap water	200	90.9
Public tap	14	6.4
Access to electricity		
Yes	220	100
No	0	0
Access to refrigerator		
Yes	132	60
No	88	40
Access to toilet		
Yes	220	100
No	0	0
Who (type of caregiver) prepared the bottle food		
Mother	188	85.5
Father	6	2.7
Sibling	13	5.9
Housemaid	5	2.3
Grandparents	8	3.6
Source of bottle food preparation information		
Family or friends	161	73.2
Health provider	34	15.5
PIF tin label	25	11.4
Hand washing prior to bottle food preparation		
No, I don’t wash	103	46.8
Yes, with water only	29	13.2
Yes, with water and soap	88	40
Water used for the preparation of bottle food		
Tap water	154	70
Boiled water	42	19.1
Bottled water	9	4.1
Public tap water	13	5.9
Home filtered water	2	0.9
Bottle food storage temperature		
At ambient temperature	137	62.3
Refrigerator temperature	83	37.3
Storage duration of prepared bottle food		
< 6 h	109	49.5
> 6 h	101	50.5
Fate (handling) of leftover		
Reuse	120	54.5
Discard	94	42.7
Consume by other children	6	2.7
Number of interchangeable bottles		
No extra bottle	96	43.6
Two bottles	63	28.6
Three bottles	61	27.7
More than three	0	0
Method of bottle washing practice		
Rinsing with plain water only	102	46.4
Washing using water and soap	74	33.6
Washing using water, soap, and brush	44	20
Frequency of washing the feeding bottle		
Once a day	94	42.7
Twice a day	46	20.9
Three times a day	41	18.6
Before/after each feed	39	17.7
Methods of bottle and nipple sterilization		
Boiling for ten minutes	98	44.5
Rinsing or soaking with/in boiled water	88	40
Never disinfect/sterilize	34	15.5

### Baby feeding bottle food product category and hygiene practices

**Fig. 1:**
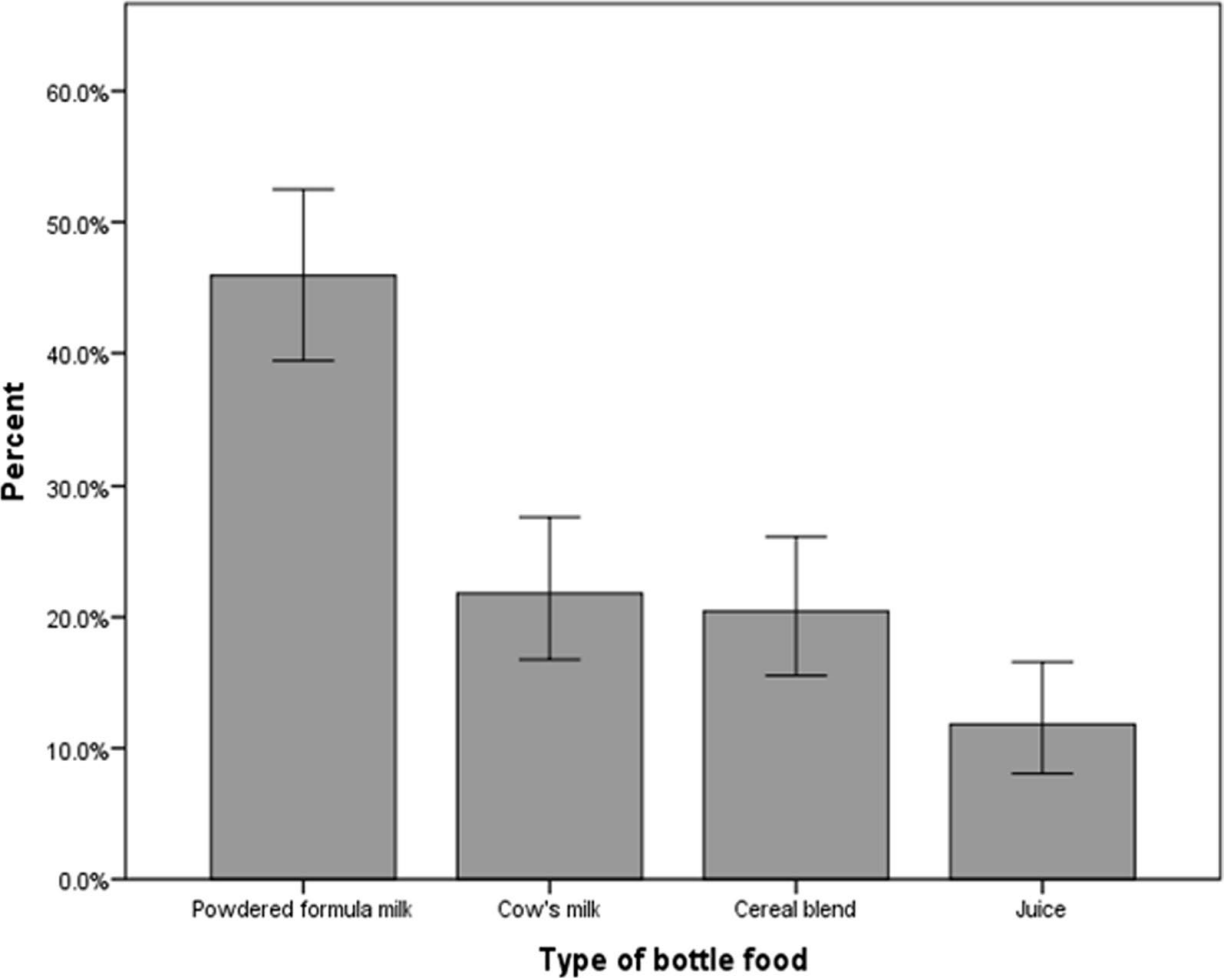
Bar chart showing the type of baby bottle foods commonly used in Arba Minch, southern Ethiopia 2022 (*n* = 220)

The contents of the baby bottles were categorized into four food types: PIF, cow milk, cereal blend, and fruit juice. One hundred and one of the caregivers, i.e., 44.9%, fed their babies PIF, 48 (21.8%) cow milk, 45 (20.5%) cereal blends, and 26 (11.8%) fruit juice (Fig. 1).

All participants responded positively when asked if they were familiar with baby food preparation and handling procedures. The majority of caregivers, i.e., 161 (73.2%), obtained the information from family members or friends, while the rest, 34 (15.5%) and 25 (11.4%), respectively, received it from a health practitioner and manufacturer guidelines found on infant formula tins (Table 1). About eighty-eight (40.0%) mothers/caregivers wash their hands at all critical time using soap and running water, and 29 (13.2%) reported washing their hands only with water. At the time of sample collection, the majority, i.e., 154 (70.0%) of the respondents, reported using tap water to prepare baby food. About 120 (54.5%) of caregivers used only a single feeding bottle, 137 (62.3%) reused the leftover bottle contents for the re-preparation of food, and half of them, 111 (50.5%) kept the prepared food at the ambient temperature for more than 6 h. The mother prepared most of the baby's food (70.8%). The most common method employed to sterilize and disinfect bottles and nipples was to boil them for at least ten minutes (44.5%), but less than half of caregivers reported boiling the bottle and nipple of baby feeding bottle once a day (42.7%). However, 15.5% of caregivers never sterilize bottles and nipples via boiling (Table 1).

### Microbiological quality and safety of baby bottle food

Out of the 220 food samples examined for the TVC, growth was detected in 188 (85.45%) bottle foods. The overall mean TVC of baby feeding bottle food content was 5.35 ± 2.3 log_10_ CFU/mL. The mean TVC for fruit juice was 6.54 log_10_ CFU/mL (95% CI 5.92–7.16) and a range of 1.0–7.72 log_10_ CFU/mL, while the mean TVC of cereal blends was 6.68 log_10_ CFU/mL, with a 95% CI of 6.17–7.18 and a range of 1.0–8.18 log_10_ CFU/mL. Fruit juice had the highest mean TVC, 6.5 log_10_ CFU/mL, with a 95% CI of 5.9–7.1 and a range of 1.0–7.7 log_10_ CFU/mL, followed by cereal blends, 6.6 log_10_ CFU/mL, with a 95% CI of 6.1–7.1 and a range of 1.0–8.1 log_10_ CFU/mL. The lowest mean TVC was observed in the case of PIF, 4.4 ± 2.5 log_10_ CFU/mL, with a 95% CI of 3.9–4.9. Out of the total food samples tested, only 35.5% (*n* = 77) were within the microbiologically acceptable level of TVC (< 10^4^). Powdered infant formula had the highest level of acceptable TVC values (52%), while fruit juice and cereal blends had the lowest, accounting for only 11.5 and 8.9%, respectively (Table 2).

**Table 2:** Quantitative evaluation of microbial quality by TVC and TCC and foodborne pathogens in food samples collected from bottle-fed babies attending health institutions in Arba Minch, southern Ethiopia, 2022 (*n* = 220)

Samples type	Microbial quality indicators								Foodborne pathogens
	Total *n* (%)	TVC (log_10_ CFU/mL)				TCC (log_10_ CFU/mL)			Positive *n*%
		Mean ± SD	Range	95% CI	*n* (%) of Acceptable	Mean ± SD	Range	95% CI	
Powdered infant formula	101 (45.9)	4.4 ± 2.5	1.0–8.0	3.9, 4.9	53 (52.5)	3.1 ± 2.5	1.0, 7.1	2.5, 3.6	3 (2.97)
Cow milk	48 (21.8)	5.4 ± 1.9	1.0–8.1	4.8, 5.9	17 (35.4)	3.7 ± 2.5	1.0, 7.2	3.0, 4.5	4 (8.33)
Cereal blend	45 (20.5)	6.6 ± 1.6	1.0–8.1	6.1, 7.1	4 (8.9)	5.7 ± 1.8	1.0, 7.1	5.1, 6.2	11 (24.0)
Fruit juice	26 (11.8)	6.5 ± 1.5	1.0–7.7	5.9, 7.1	3 (11.5)	5.7 ± 1.9	1.0, 7.3	4.9, 6.5	1 (3.84)
Total	220	5.3 ± 2.3	1.0–8.1	5.0, 5.6	77 (35.5)	4.1 ± 2.6	1.0, 7.3	3.7, 4.4	19 (8.63)

*PIF* powdered infant formula, *SD* standard deviation, *CI* confidence interval, *TVC* total viable count, *TCC* total coliform count

In this study, the prevalence of coliform-positive food samples was 60.5% (133/220). The mean coliform count was 4.1 ± 2.6 log_10_ CFU/mL, with a 95% CI of 3.7 to 4.4 ranging from 0 to 7.3 log_10_ CFU/mL. Coliform bacteria were below the detection limit in 39.5% of the food samples. The mean TCC recorded in cereal blends and fruit juice was the highest, 5.7 ± 1.8 log_10_ CFU/mL and 5.7 ± 1.9 log_10_ CFU/mL, respectively. The mean TCC in cow milk was 3.7 ± 2.4 log_10_ CFU/mL, showing variability in the samples tested. About 60.5% of the baby food samples had a mean TCC exceeding the maximum limits set for an acceptable level of microbiological quality (Table 2).

In this study, the overall prevalence of foodborne pathogens was 8.6% (19/220), making these food samples potentially hazardous for human consumption. The ranking order of foodborne pathogen recovery in weaning food from the baby feeding bottle was as follows. Cereal blends (24%), fruit juice (3.84%), cow milk (8.33%), and powdered infant formula (2.97%) (Table 2).

#### Microflora analysis of baby bottle foods

**Fig. 2:**
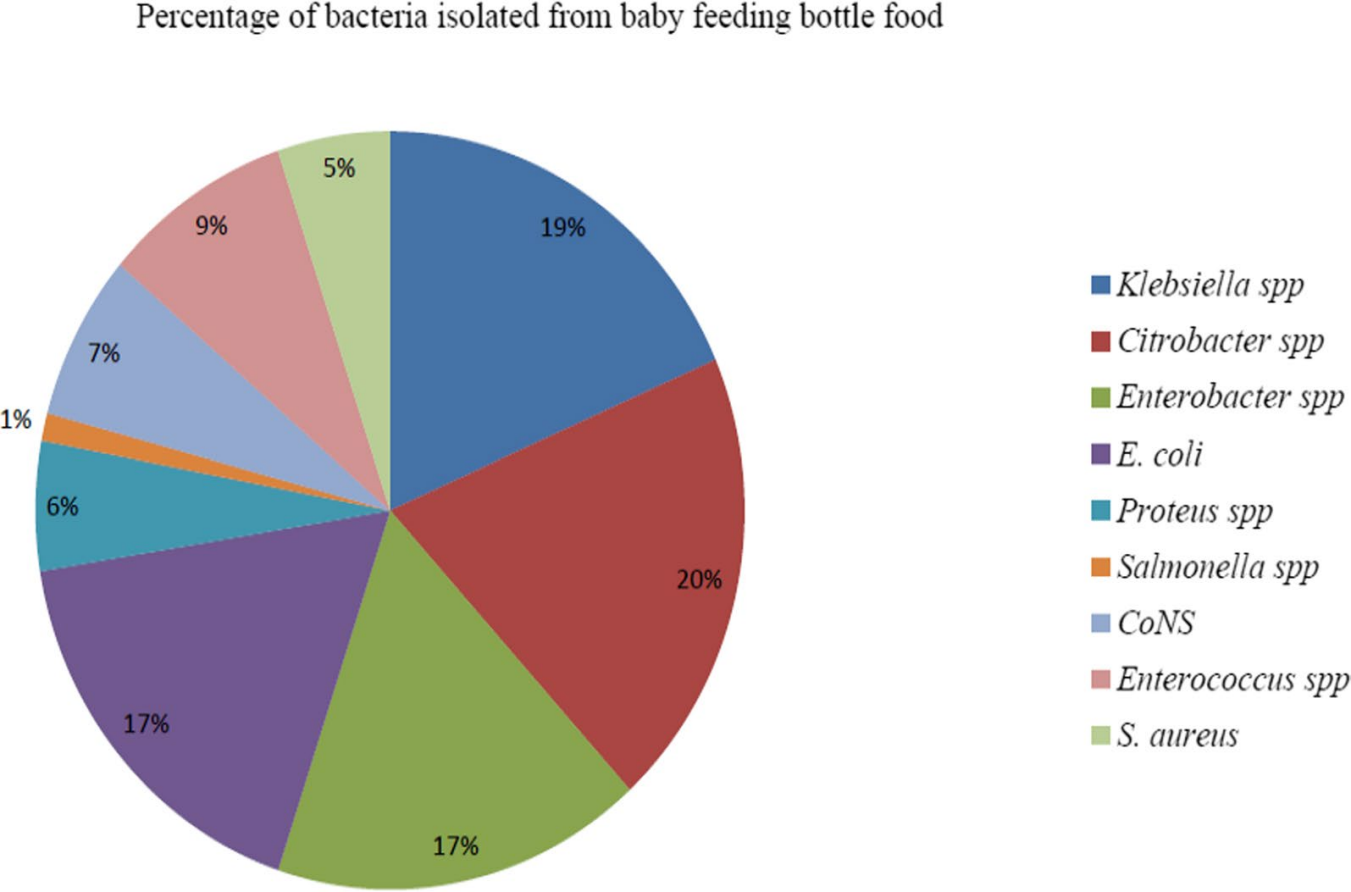
A pie chart showing the bacterial profiles versus baby bottle foods in Arba Minch, southern Ethiopia 2022 (*n* = 254)

Based on the morphological and biochemical characteristics, 254 bacteria were isolated from 220 samples. Of the total Gram-negative bacilli, isolates belonging to the family of *Enterobacteriaceae* were the most frequently isolated bacteria accounting for 79.1%, followed by Gram-positive cocci (20.8%). Among the isolates of bacteria, *Citrobacter* spp. (*n* = 50) was predominant, followed by *Klebsiella* sp. (*n* = 47), *E. coli* (*n* = 44), and *Enterobacter* spp. (*n* = 43). The least frequent bacteria were *Salmonella* spp. (*n* = 3) (Fig. 2).

##### Distribution of bacteria by food type

The cereal blends had the highest bacterial yield, accounting for 31.1%. In contrast, the juice samples contained the least microflora, i.e., 19.2%. The bacterial species isolated from the four categories of food samples were also varied. For example, Salmonella spp. was detected only in cow milk and cereal blend samples, while Proteus spp. was isolated from cereal blends and fruit juice only. Moreover, Enterococcus spp. was only detected in the fruit juice samples (Table 3).

**Table 3:** Bacterial profile of food of bottle-fed babies attending health institutions in Arba Minch, southern Ethiopia, 2022 (*n* = 220)

Food type	Bacterial profile
Powdered infant formula	*Klebsiella* spp., *Enterobacter* spp., *Citrobacter* spp., *E. coli* CoNs*, Enterococcus* spp., *S. aureus*
Cow milk	*E. coli, Enterobacter, Salmonella* spp., *Citrobacter* spp., *Klebsiella* spp., CoNs*, Enterococcus,* and *S. aureus*
Cereal blend	*E. coli, Enterobacter* spp., *Proteus* spp., *Salmonella* spp., *Citrobacter* sp., *Klebsiella* spp., CoNs*, Enterococcus* spp., and* S. aureus*
Fruit juice	*E. coli, Enterobacter* spp*., Proteus* spp., *Citrobacter* spp., *Klebsiella* spp., CoNs and*, S. aureus*
Total	*Enterobacteriaceae* (*n* = 201)
	Gram-positive cocci (*n* = 53)

### Relationship between TCC and other variables

The associations between TCC and the demographics and hygienic characteristics of caregivers or mothers were assessed using a one-way ANOVA. The results revealed that microbial contamination of bottle food with respect to the TCC was significantly associated with the types of food and food hygiene practices of mothers/caregivers. The study found a statistically significant association between contamination with coliform bacteria and the type of bottle food. Accordingly, the mean TCC recorded for the PIF (mean TCC = 3.1 log_10_ CFU/mL) was significantly lower than that obtained from the cereal blends (mean TCC = 5.7 log_10_ CFU/mL, *p* = 0.001) and in fruit juice (mean TCC = 5.7 log_10_ CFU/mL, *p* = 0.001). However, there was no statistical difference detected between the TCC determined in the cases of PIF and cow milk (mean TCC = 3.7 log_10_ CFU/mL, *p* = 0.36). Furthermore, no significant difference was detected between TCC in cereal blends and fruit juice (*p* > 0.05). Although there was no statistically significant difference in the microbial load of bottle food in terms of the sex of the caregivers (*p* = 0.69), the relation of the caregivers to the baby (*p* = 0.66), marital status (*p* = 0.09), and the person who prepared the bottle food (*p* = 0.69), lower TCC values (1.5 log_10_ CFU/mL) were recorded in food samples obtained from those caregivers who had a diploma and above. The mean TCC was significantly different for the food samples obtained from illiterate caregivers who could read and write only and those who had completed primary school and high school education. The source of information on bottle food preparation and handling was assessed in relation to contamination, and the mean TCC showed a significant difference in the three groups. The highest mean TCC was found in the food samples collected from the caregiver who prepared and handled the baby bottle based on information they received from family or friends (TCC = 4.9 log_10_ CFU/mL) as compared to those whose source of information was a health practitioner (TCC = 2.0 log_10_ CFU/mL) and those who prepared the bottle according to the instructions provided on the guideline (TCC = 1.8 log_10_ CFU/mL). These differences were statistically significant (*p* = 0.001). However, the contamination load did not differ among caregivers who used health practitioners and guidelines as a source of information (*p* = 0.9) (Table 4).

**Table 4:** ANOVA for total coliform count (TCC log_10_ CFU/mL) in bottle foods among bottle-fed babies attending health institutions in Arba Minch, southern Ethiopia, 2022 (*n* = 220)

Variable	Total (*n*)	TCC log_10_ (CFU/mL)				
		Mean TCC	SD	*DF*	*F* statistic	Sig
Age of caregivers/mothers						
< 20	8	3.6046	2.80365	(3, 216)	11.507	0.069
20–30	130	4.8977	2.35425			
31–40	70	2.8611	2.50385			
> 40	12	3.0620	2.70357			
Educational status						
Illiterate	56	5.8642^c^	1.68984	(4, 96.9)	51.364	0.001
Reading and writing only	28	5.6636^c^	1.73488			
Primary school	52	4.5231^c^	2.49690			
Secondary school	38	2.8765^b^	2.34846			
College/university	46	1.5449^a^	1.62228			
Relation to the babies						
Mother	168	4.1695	2.60058	(4, 215)	0.593	0.733
Father	30	3.8773	2.55653			
Sibling	11	4.0646	2.96320			
Housemaid	4	2.3253	2.65051			
Grandparents	7	4.5349	2.70671			
Type of bottle food						
Powdered formula milk	101	3.1091^a^	2.58361	(3, 87.05)	21.447	0.001
Cow milk	48	3.7770^a^	2.49231			
Cereal blends	45	5.7405^b^	1.80137			
Juice	26	5.7277^b^	1.91476			
Source of information on the preparation of bottle food						
Family or friends	161	4.9002^a^	2.37182	(2, 45.87)	54.150	0.001
Healthcare provider	34	2.0014^b^	1.95183			
PIF tin label	25	1.8234^b^	1.82384			
Hand washing practice before baby bottle food preparation						
No, I don’t wash	103	6.1968^a^	0.95563	(2, 64.68)	270.68	0.001
Yes, with water only	29	4.1213^b^	2.58610			
With water and soap	88	1.6451^c^	1.60622			
The main way of dealing with already prepared leftovers						
Reuse it for the next day	120	5.6905	1.78189	(2, 13.35)	89.263	0.001
Discard	94	2.0624	2.02831			
Consume by other children	6	4.3060	2.58853			
Storage temperature						
Ambient temperature	137	5.5969	1.83452	(1, 188.5)	276.245	0.001
Store in refrigerator	83	1.6359	1.63563			
Storage duration						
0–2 h	68	1.8664^a^	1.88065	(3, 105.3)	119.10	0.001
3–5 h	41	2.9183^b^	2.52523			
6–12 h	86	5.8083^c^	1.63205			
24 h and above	25	6.2593^c^	0.48123			
Number of exchanging bottle						
No extra bottle	96	6.0921^a^	1.23947	(2, 112.1)	179.539	0.001
Two bottles	63	3.4962^b^	2.54108			
Three bottles	61	1.5976^c^	1.61165			
Bottle cleansing and washing						
Rinsing with plain water only	102	5.4876^a^	1.93197	(2, 127.40)	115.03	0.001
Washing using water and soap	74	3.8351^b^	2.62358			
Washing using water, soap, and brush	44	1.3415^c^	1.29559			
Frequency of washing the feeding bottle						
Once a day	94	6.0433^a^	1.32023	(3. 86.58)	102.79	0.001
Twice a day	46	4.1818^b^	2.43821			
Three times a day	41	2.0109^c^	2.00055			
Before/after each feed	39	1.5301^c^	1.67441			
Method of bottle and nipple sterilization						
Boiling for ten minutes	98	1.6266^a^	1.57668	(2, 133.08)	303.17	0.001
Rinsing or soaking with/in boiled water	88	6.0901^b^	1.31440			
Never disinfect/sterilize	34	6.0947^b^	0.63332			

^a,b,c^Statistically significant difference down a group but no statistical significance difference in letters. *NA* not applicable because assumptions are not fulfilled

### Factors associated with the microbial load of baby bottle food

Multi-linear regression analysis was performed for all independent variables, with the final model suggesting that three independent variables had a significant influence on the total coliform count on baby bottle food samples.

Examination of the regression coefficient indicates that sterilization and disinfection of the baby feeding bottle has a significant effect on a load of total coliform bacteria (*p* < 0.001), and in this case, the standard correlation coefficient was found to be positive [*t* = 7.8, *β* = 0.7, 95% CI 0.5, 0.9]. As a result, for each unit that mothers or caregivers fail to sterilize or disinfect the feeding bottles, there is a beta weight increase in a load of total coliforms on the baby feeding bottle food of 0.7 log_10_ CFU/mL (Table 5).

**Table 5:** Multiple linear regression analyses of factors associated with TCC in bottle foods among bottle-fed babies attending health institutions in Arba Minch, southern Ethiopia, 2022 (*n* = 220)

Dependent variable	Predictors	Unstandardized coefficients		Standardized coefficients (beta)	*t*	Sig	95% CI
		*β*	SD				
TCC log _10_ CFU/mL	Constant	3.244	0.670		4.842	0.000	1.92–4.56
	What method of sterilization of bottles do you use?	0.764	0.097	0.488	7.882	0.000	0.57–0.95
	Hand washing prior to handling & preparation of bottle foods	0.838	0.173	0.299	− 4.85	0.000	1.17–0.498
	Type of baby feeding bottle foods	0.406	0.085	0.166	4.788	0.000	0.24–573

*TCC* total coliform count, *SD* standard deviation

Similarly, examination of the regression coefficient reveals that the type of food in a baby feeding bottle has a significant effect on the TCC of baby feeding bottle food content at the time of consumption [*t* = 4.8, *β* = 0.4, 95% CI 0.2, 57, *p* < 0.001]. Based on the beta weight, the direction of the impact is positive, and for each unit increase in the use of cereal blends or fruit juice in a baby feeding bottle, there is a beta weight of 0.40 log_10_ CFU/mL increase in TCC.

Furthermore, multiple linear regression analysis results reveal that the hand washing practice of caregivers or mothers prior to the preparation of baby food has a significant impact on the total coliform count on the baby feeding bottle food (*p* < 0.001). Based on the beta weight, the direction of the impact is negative. Therefore, for each unit increase in hand washing, there is a beta of 0.83 log_10_ CFU/mL decrease in the load of TCC [*t* = 4.8, *β* = 0.83, 95% CI − 1.17, − 0.49]. Based on the relative strength of the beta weights, washing practice [*β* = 0.83, *p* < 0.001] was shown to be the most statistically significant of all the predictive variables to TCC on food samples (Table 5).

## Discussion

This study aimed to elucidate the microbiological quality and food safety of bottle food collected from children under five attending three health institutions in Arba Minch, southern Ethiopia. To the best of our knowledge, this study is the first to provide unique insights into the microbial quality and safety of bottle food in the study area. In addition, the study was designed to identify significant differences in contamination versus socioeconomic characteristics, knowledge of food safety, and food safety practices.

The overall findings of this study revealed that 57.3 and 60.5% of the food samples had bacterial counts above the maximum standard values set for TVC and TCC, respectively, and were therefore deemed unacceptable in terms of microbial quality. In addition, approximately 8.6% (*n* = 19) of the samples contained foodborne pathogens and were considered potentially hazardous if consumed. The overall microbial contamination of baby feeding bottle food in this study was in line with food safety risk highlighted in previous study from Gambia in which contamination ranges from 5 to 70% [14], Kenya (14.4 to 62% of contamination) [19], Nigeria (microbial load ranges from 3.3 to 5.02 log_10_ CFU/mL [12, 17], Thailand [20], and Bangladesh in which the rate of contamination was estimated as 41% [9, 34, 35] and Ethiopia (contaminations ranges from 14.2% and 31.2%) [16].

The aerobic mesophilic bacteria among baby feeding bottle foods were estimated to be in the range of 1.0 to 8.1 log_10_ CFU/ml. The finding was in line with the results of previous studies conducted in Ethiopia (7.29 log_10_ CFU/mL) [16], Nigeria (5.02 log_10_ CFU/mL) [17], and Korea (2–5 log_10_ CFU/mL) [18]. This value point out that the baby bottle food in the current study area supported the growth of microorganisms due to a conducive environment [36] suggesting the high risk of microbial spoilage in baby feeding bottle foods particularly cereal blend and fruit juice samples.

On the one hand, the overall mean TVC in the food samples was 5.3 ± 2.3 log_10_ CFU/mL, which was lower than the findings reported in a couple of studies done in Ethiopia (8.07 log_10_ CFU/mL) [32], and Thailand (6 log_10_ CFU/mL in about ten percent of the samples) [20]. In comparison to the study conducted in Ethiopia, the lower count observed in the current study may be partly explained by the time in which these studies were conducted and the improvement in attitude and practice of child feeding practices, while socioeconomic and geographical differences may explain the differences from the study conducted in Thailand. On the other hand, it exceeds the value previously reported in a study done in Brazil [6]. Variations may be attributed to differences in food characteristics, caregiver knowledge and practices, and sociodemographic factors. Our study revealed that about 60.5% of the food samples were potentially contaminated with feces, which is vis-à-vis the results of a series of previously published studies [14, 16, 19, 20]. Moreover, the reported load of microbial contamination was higher than the earlier study done in Korea (2–5 log_10_ CFU/mL) [18]. The presence of TCC higher than the permissible level set for the acceptable quality standard of ready-to-eat foods has been typically used to measure the effectiveness of disinfection and to indicate the possible presence of enteric bacterial pathogens [37].

Consequently, 59.1% of the food samples in this study with TCC above the maximum allowable limit (> 10^2^) suggest an unhygienic processing of baby feeding bottle food is common in the study area. Furthermore, the finding also suggest the source of contamination of complementary food in baby feeding bottle could be more likely to be feaces contamination as indicated in previous study [8]. However, this observation can also be explained by the fact that improper human practices can cause extrinsic contamination during food processing, handling, and storage or as a result of using contaminated water. In addition, the high level of TCC detected in our study suggests that the equipment and utensils used to prepare and store baby bottle food must be subjected to rigorous hygiene and cleaning procedures.

The proportion of microbiologically unacceptable quality of bottle food using the TCC parameter was 60.5%, and this was lower than the results reported from South Africa (84.6%) [28], Indonesia (88%) [31], and Thailand (66%) [38]. The observed variations in the contamination levels may be attributed to differences in the food type, sample size, the time of the study conducted, laboratory protocols employed, cleaning and disinfection practices, bottle and nipple sterilization practices, sociocultural characteristics, baseline prevalence of foodborne pathogens, seasonality, and geographical location [17].

In the present study, a high proportion (i.e., 79%) of Gram-negative bacteria belonging to the family *Enterobacteriaceae* contaminated food samples at the point of consumption. The result validates the conclusion of a previous study conducted in Jimma, Ethiopia [22]. The predominant microflora of the food sample in this study was *Citrobacter* sp., followed by *Klebsiella* spp. and *E. coli*. The presence of these bacteria in the samples denotes that the food had been handled and stored in a poorly hygienic manner [25] and could also be linked to cross-contamination in the food processing chain or from food handlers. Furthermore, the higher isolation rates of members of the *Enterobacteriaceae* family and the Gram-positive Staphylococci (*Enterococcus* spp. and *S. aureus*) intimate that bottle food may serve as a vector for the transmission of both pathogenic and opportunistic pathogens. The findings agree with similar studies done in Pakistan [7] and Nigeria [12, 17, 21].

The hygiene criteria tested in this study, such as TVC, TCC, and *Enterobacteriaceae*, per se, indicate the possibility of fecal contamination of food due to poor hygiene practices, insufficient processing, and storage [6, 15]. Additionally, the findings indicate non-compliance with food hygiene criteria, necessitating corrective actions, such as implementing food safety measures during food production [9, 17, 33].

The higher percentage of contaminated food samples with *Enterococcus* spp., *S. aureus, Salmonella* spp., and diarrheagenic *E. coli* was another significant public health concern revealed in our study [10, 39]. Food contamination, for example, has been identified as a substantial contributor to diarrhea in low-income settings, with up to 70% of diarrheal episodes linked to pathogen-contaminated water and food [11, 15, 21]. Furthermore, the detection of *Salmonella* spp., *S. aureus,* and diarrheagenic *E. coli* in 8.5% of food samples at the time of consumption reflects the magnitude of exposure to the foodborne pathogens among bottle-fed babies in the study settings [10, 34].

In addition, the prevalence of skin and mucosal microflora on baby bottle food samples may indicate contamination by food handlers after preparation or during handling practices [24]. However, the isolation of *S. aureus* rate in the current study was lower than the results reported from two cities in Ethiopia [22, 37] and Sudan [40]. The results specify the importance of improved hand washing and sterilization of feeding bottles by caregivers. The isolation rate of Salmonella spp. and diarrheagenic *E. coli* in the food samples was only 1.36% (3/220). However, a higher rate of Salmonella spp. was reported from studies done in other Ethiopian cities, Jimma [22] and Addis Ababa [37]. The low-level detection of diarrheagenic *E. coli* (1.36%) in food samples in our study is in parity with the results reported from Thailand (1.2%) [38] and Ethiopia (3.7%) [32]. At the same time, it is lower than the results reported elsewhere (61.4%) [41]. However, the detection level of the foodborne pathogens (*Salmonella* spp. and *S. aureus*) sharply contrasts with an earlier study [18]. Interestingly, *Shigella* spp. was not detected in the food samples tested in this study. Similarly, *Shigella* spp. is not recovered in food samples in other studies conducted in Thailand [38], Ethiopia [37] and Egypt [33, 42]. According to previous study *Shigella* spp. are not as persistent as *Salmonella* spp. or are  most likely eliminated during cooking [41, 43]. However, studies from Ethiopia [32], Sudan [40], and India [44] successfully recovered *Shigella* spp. in baby foods. These fluctuations may also be attributed to differences in the type of food ingredient and food source, the water level of the food, the level of compliance with the standard preparation and handling techniques, and other environmental factors [36].

There is considerable evidence that shows diverse organisms can multiply rapidly on food and are relatively resistant to routine cleansing and disinfection. Therefore, without additional risk-based and decisive measures to improve food hygiene and safety practices, the burden of foodborne disease in children could not be controlled [10, 12, 20]. Accordingly, we identified the type of supplementary food, mothers’ or caregivers’ source of information for food preparation, hand washing practices before food preparation, the type of water used for baby food preparation, the handling of leftover baby food, storage conditions (temperature and duration), as well as sterilization and disinfection of the feeding bottle as critical control points using ANOVA. The observations suggest systematic planning and management of food hygiene practices in the study setting. Furthermore, the identified factors are indicators of the critical control points to implement food safety measures that can reduce exposure risk and improve children’s health outcomes [6, 17, 29, 31].

In this study, after adjusting all the confounding factors, the types of baby feeding bottle food, caregiver hand washing practices before food preparation, and methods of bottle sterilization and disinfection were identified as independent predictors of the microbial quality of home-prepared baby bottle food. The identified factors were broadly consistent with the results of a couple of other quantitative studies done elsewhere [17, 26]. In this study, the TCC on the baby feeding bottle food was found to be significantly related to the types of bottle food [*β* = 0.4 (95% CI 0.2–0.5), *p* < 0.001]. The mean TCC in the cereal blends or fruit juice was significantly higher than that of PIF and cow milk by 0.41 log_10_ CFU/mL. The type of food was also associated with microbial quality (load) in a prior study conducted elsewhere [15]. The current finding shows that babies who consume cereal blend or fruit juice foods in feeding bottles are at higher risk of being exposed to foodborne pathogens than those who use powdered infant formula [18, 34]. The high bacterial count observed in the cereal blends could be attributed to inadequate heat penetration during its preparation or contamination that occurred after cooking from the hands of the caregiver or feeding bottle [18]. The high load of coliform contamination in fruit juice in this study indicates this is a global problem, as several other studies have also revealed the high level of TCC in fruit [8, 9, 24, 35]. In the current study, significantly low levels of coliform bacteria were observed among the foods sampled from caregivers who habitually sterilized the feeding bottles (*p* < 0.001). The linear regression model results revealed that the TCC increased by 0.7 log_10_ CFU/mL each time mothers or caregivers did not sterilize or disinfect baby feeding bottles. This observation resembles the results of previous studies done in Nigeria [17], South Africa [13], Mali [10], Kenya [39], Brazil [6], and elsewhere [24]. The finding provides a fascinating insight into the potential control measures for contamination and ensuring food safety in childcare practices [45]. However, studies from Brazil and UK failed to demonstrate the relationship between median TCC versus washing or disinfection practices [6, 46]. Additionally, a previous study could not detect a significant correlation between cleaning methods and bacterial contamination of foods [47].

On the other hand, hand washing by the caregiver or mother before handling and preparing food was associated with a lower risk of contamination [*β* = 0.8, 95% CI 1.1–0.4). While preparing baby bottle food, every additional hand wash decreases the mean TCC by 0.8 log_10_ CFU/mL (*p* 0.0001) (Table 5). The findings confirmed that the protective effect of practicing good hand hygiene during food preparation is a critical control point to reduce the contamination rate [9, 48].

### Implications of the findings

The findings have implications for current baby feeding methods and childcare practices. The high microbiologically unacceptable quality and unsafe food for consumption in supplementary baby food in this study suggest that bottle feeding practice should be considered with all precautions since the food in baby feeding bottles supports the growth of different indicators and pathogenic bacteria. The critical point of baby supplementary food contamination and the pathogens identified in this study should be used for evidence-based decision-making and to improve childcare practices for reducing diarrhea caused by the most common enteric pathogens. Improved bottle feeding practice would include multi-disciplinary action in planning and implementing an effective intervention to prevent and control pathogen transmission through supplementary food. This could be accomplished by improving education about bottle feeding practices. They are promoting alternate feeding methods, better cleaning of utensils, sterilization of baby feeding bottles, and improved preparation techniques by increasing awareness of caregivers and mothers on the microbial quality and safety of supplementary foods and educating them on the best practices of washing, preparation, handling, and storage of supplementary nutrition, as well as the country-wide implementation of microbial monitoring and evaluation of supplementary foods under the principle of hazard analysis and critical control point. The estimates could be used as inputs by government sectors, non-governmental organizations, and policymakers to enforce food safety and childcare regulations. The finding can be used as a baseline for future studies. Large-scale research using molecular techniques would be required to prove the transmission of bacterial diseases from supplementary food via bottle feeding practice. Further work is warranted to provide a complete picture of the extent of the contamination of other types of supplementary foods in the community and to assess the risk of prevalent foodborne diseases. Given that bottle feeding of baby is the common practice throughout low-income countries and the common type of complementary food used is also predominantly similar as it was recommended by the health care professional and WHO guideline, the food safety risk identified in this study can be generalizable for other similar setting especially in low-income countries. In addition to this, food quality and food safety are the priority agenda in reduce child morbidity and mortality. As food microbial contaminations are continued to contribute for the high burden of diarrhea, the finding from this study will interest   the global effort planning in controlling the foodborne disease especially among the most vulnerable group of population such as children less than five years of age.

### Limitations

Although this study covered essential issues, it has some limitations. First, this study relied on self-reported data on bottle handling and hygiene practices, which may have resulted in overestimating positive bottle handling and hygiene practices. The second limitation relates to this study was spectrum of foodborne bacterial pathogens were not studied using molecular techniques and pathogens with special isolation requirements, such as *Campylobacter* spp. and *Bacillus cereus* were not included. In this study, we could not determine the seasonal trend or impact of contamination. Similarly, the risk factors associated with additional food contamination along the production chain were not addressed. Finally, the study was conducted in healthcare institutions in an urban area; therefore, the precise estimates may not apply to the community as a whole.

### Conclusions

The following conclusions are drawn based on the forgone analysis

- Food safety and quality are public health concerns in bottle feeding practices.
- More than half of baby bottle food had an acceptable microbiological quality.
- About one in ten baby foods are potentially hazardous, as indicated by the presence of foodborne pathogens.
- *Salmonella* spp., *E. coli,* and *S. aureus* were the most common foodborne bacterial pathogens in bottle food.
- Factors that independently predict microbial quality of supplementary baby food include food type, (cereal blend and fruit juice are high-risk food), hand hygiene practice, sterilization and disinfection of feeding bottles.

## Supplementary Information

**Additional file 1: Table S1.** Questionnaire.

**Additional file 2.** **Table S2:** Microbiological limits for Infant food and ready-to-eat food).

**Additional file 3.** STROBE checklist.

## Data Availability

All the data used and/or analyzed in this study are presented, and data will be available from the corresponding author upon reasonable request.

## Abbreviations

CFU: Colony forming unit
ISO: International Organization for Standardization
SPSS: Statistical Package for the Social Sciences
TCC: Total coliform count
TVC: Total viable count
WHO: World Health Organization
