# Supplementary material for: Bacteriological quality and safety of bottle food and associated factors among bottle-fed babies attending pediatric outpatient clinics of Government Health Institutions in Arba Minch, southern Ethiopia
Source: J Health Popul Nutr. 2023 May 26;42:46. doi: 10.1186/s41043-023-00387-1 (PMC10214617; doi:10.1186/s41043-023-00387-1)
Supplement: Supplementary file 1 — Additional file 1: Table S1. Questionnaire. [file 41043_2023_387_MOESM1_ESM.docx]

Questionnaire English version

**Questionnaire number______________ Date of interview:** _**_______________**

**Card number: _____________________ Data collector name: ______________**

**Section One: Socio-demographic characteristics of caregivers**

1. Age of the caregiver________________
2. Gender of caregiver ________________
3. Marital status
   1. Married
   2. Unmarried
   3. Divorced
   4. Widowhood
4. The occupation of the caregiver
   1. Housewife
   2. Merchant
   3. Farmar
   4. Labor
   5. Governmental employee
   6. Other (mention)
5. The role of caregiver to the baby
   1. Mother
   2. Father
   3. Siblings
   4. Housemaid
   5. Other(mention)
6. Highest completed educational level of caregiver
   1. Illiterate
   2. Reading and writing only
   3. Primary school
   4. Secondary school & above
   5. College/University
7. Monthly income level of the family? ______________________
8. Source of drinking water supply?
   1. Well water
   2. Tap water
   3. Spring water
   4. Bottled water
   5. Other (mention)
9. Do you have electricity supply in your house?
   1. Yes
   2. No
10. Do you own refrigerator in your house?
    1. Yes
    2. No
11. Do you have toilet
    1. Yes
    2. No

**Section two: Demographic characteristics of the baby**

1. Sex of the baby? _______________________
2. Age of the baby? ________________
3. The reason for today’s hospital visit? _____________________
4. The age bottle feeding is commenced? ____________________

**Section Three: bottle food preparation, storage, and bottle hygiene practices**

1. What is the constituent of the bottle content?
   1. Formula milk
   2. Natural cow’s milk
   3. Cereal blend
   4. Fruit juice
   5. Other (specify)
2. Who prepared the bottle food?
   1. Mother
   2. Father
   3. Siblings
   4. Housemaid
   5. Other (mention)
3. Primary source of information of bottle food preparation?
   1. Family and friends
   2. Health practitioner
   3. Manufacturer instruction on infant formula tin
   4. Other (mention)
4. Do you wash your hand before the preparation of the bottle food?
   1. No
   2. With water only
   3. With soap and water
5. Water used for preparing feeding bottle?
   1. Tap water
   2. Boiled water
   3. Bottled water
   4. Other (mention)
6. How long you keep the bottle food after preparation?
   1. 0-2 hrs.
   2. 3-6 hrs.
   3. 6-12 hrs.
   4. 24 hrs. and above
7. How do you store the bottle food after preparation?
   1. Stored in ambient temperature
   2. Store in refrigerator
   3. Other (mention)
8. Fate of the bottle left over content?
   1. Reuse
   2. Discard
   3. Consume by mother or another child
   4. Other (mention)
9. How many interchangeable feeding-bottles do you have?
   1. No extra bottle
   2. Two
   3. Three
   4. More than three
10. How do you clean used bottle and its accessory?
    1. Rinsing with water only
    2. Washing with soap and water
    3. Washing with soap, brush, and water
    4. Other (mention)
11. How often do you wash the feeding bottle and nipple?
12. Once daily
13. Twice daily
14. Three times daily
15. After every feed/use
16. What method of sterilization of bottle you use?
    1. Boiling for ten minutes
    2. Soaking in sterilizing solution
    3. Electric sterilizer or microwave
    4. Other (mention)
