# Supplementary material for: Bacteriological quality and safety of bottle food and associated factors among bottle-fed babies attending pediatric outpatient clinics of Government Health Institutions in Arba Minch, southern Ethiopia
Source: J Health Popul Nutr. 2023 May 26;42:46. doi: 10.1186/s41043-023-00387-1 (PMC10214617; doi:10.1186/s41043-023-00387-1)
Supplement: Supplementary file 2 — Additional file 2. Table S2: Microbiological limits for Infant food and ready-to-eat food). [file 41043_2023_387_MOESM2_ESM.docx]

Additional information,

Supplementary table S2: Microbiological limits for Infant food and ready-to-eat food

| Food category | Test method | Result: colony forming unit (CFU/g or CFU/ml) | | | |
| --- | --- | --- | --- | --- | --- |
|  |  | Satisfactory | Marginal | Unsatisfactory | Hazards |
| Milk powder, powdered dairy products, other reconstituted powdered foods ready to eat after reconstitution or warming | Aerobic plate count | $<{10}^{4}$ | ${10}^{4}-{10}^{6}$ | $\geq{10}^{6}$ | N/A |
|  | Coliform count | $\leq10$ | - | ${\geq10}^{2}$ | N/A |
|  | *Salmonella spp* | ND | N/A | N/A | Detected |
|  | *Shigella spp* | ND | N/A | N/A | Detected |
|  | Pathogenic *E coli* | ND | N/A | N/A | Detected |
|  | *S. aureus count* | <20 | 20-${\leq10}^{4}$ | 100-${<10}^{4}$ | ${\geq10}^{4}$ |
| All RTE food | Enterobacteriaceae | $<100$ | $100-{<10}^{4}$ | $\geq{10}^{4}$ | N/A |
|  | *E. coli* | <20 | 20-100 | $\geq{10}^{2}$ | N/A |
|  | *S. aureus* count | <20 | 20-${\leq10}^{4}$ | 100-${<10}^{4}$ | ${\geq10}^{4}$ |
