# Supplementary material for: Bacteriological quality and safety of bottle food and associated factors among bottle-fed babies attending pediatric outpatient clinics of Government Health Institutions in Arba Minch, southern Ethiopia
Source: J Health Popul Nutr. 2023 May 26;42:46. doi: 10.1186/s41043-023-00387-1 (PMC10214617; doi:10.1186/s41043-023-00387-1)
Supplement: Supplementary file 3 — Additional file 3. STROBE checklist. [file 41043_2023_387_MOESM3_ESM.docx]

STROBE Statement—checklist of items that should be included in reports of observational studies

|  | Item No. | Recommendation | Page  No. | Relevant text from manuscript |
| --- | --- | --- | --- | --- |
| **Title and abstract** | 1 | (*a*) Indicate the study’s design with a commonly used term in the title or the abstract | 2 | Cross sectional study is stated in the abstract line 32 |
|  |  | (*b*) Provide in the abstract an informative and balanced summary of what was done and what was found | 2 | In the abstract Background , objective, methodology , results and conclusion are included from line 33 to 51 |
| Introduction | | | |  |
| Background/rationale | 2 | Explain the scientific background and rationale for the investigation being reported | 4 to 6 | Rationale, existing research gaps and problems are well explained as  “Microbial contamination of baby bottle food has been identified as a significant public health concern, especially in developing countries, but it remains overlooked. Therefore, the purpose of this study was determine microbiological hazards, compliance with hygiene practices, and critical control points of contamination in homemade baby bottle food in southern Ethiopia. |
| Objectives | 3 | State specific objectives, including any prespecified hypotheses | 6 | It is noted in the abstract as well as at the end of the introduction as follows-  “the main objective of this study was to evaluated the bacteriological quality of baby feeding bottle foods samples using hygine indicator parameters (TVC and TCC) and assessing the food safety by detecting the common food borne pathogens in a food samples at the time of consumption. As secondary objective the study also assessed the the load of bacterial contamination in relation to participants' demographics and food hygiene practices involving mothers/ caregivers and bottle-fed babies attending three health institutions in Arba Minch, southern Ethiopia. Line 116 to 122 |
| Methods | | | |  |
| Study design | 4 | Present key elements of study design early in the paper | 7 | Study design is twice stated in the abstract as well as material and method sections as follows  “institution-based cross- sectional on page 6 line 127 |
| Setting | 5 | Describe the setting, locations, and relevant dates, including periods of recruitment, exposure, follow-up, and data collection | 7 | Details of study set-ups, study area, period, time of particpants enrolment and data collection are mentioned in the materials and method sections as follows “Three Government  Health Institutions in Arba Minch, southern Ethiopia.  From February 01 to April 30, 2022” |
| Participants | 6 | (*a*) *Cohort study*—Give the eligibility criteria, and the sources and methods of selection of participants. Describe methods of follow-up  *Case-control study*—Give the eligibility criteria, and the sources and methods of case ascertainment and control selection. Give the rationale for the choice of cases and controls  *Cross-sectional study*—Give the eligibility criteria, and the sources and methods of selection of participants | 7 | Details of the source and study population, their eligibility criteria as well as the recruitment strategies were explained in the material and methods as follows  at page 7  Caregiver/mothers –baby pairs babies with feeding bottle were recruited via systematic random sampling. |
|  |  | (*b*) *Cohort study*—For matched studies, give matching criteria and number of exposed and unexposed  *Case-control study*—For matched studies, give matching criteria and the number of controls per case |  |  |
| Variables | 7 | Clearly define all outcomes, exposures, predictors, potential confounders, and effect modifiers. Give diagnostic criteria, if applicable | 8 | Outcome variables are microbial quality and safety  Validity of all variables was previously assessed for misunderstanding potential cofounders, and the ones with clear problems were not used in any part of the analysis |
| Data sources/ measurement | 8* | For each variable of interest, give sources of data and details of methods of assessment (measurement). Describe comparability of assessment methods if there is more than one group | *8 -10* | Predictors and outcome variables are described; possible confounders and modifiers are studied |
| Bias | 9 | Describe any efforts to address potential sources of bias | 7 | We notably tried to reduce bias by excluding in eligibility  and analysis   1. Proportional allocation of samples 2. Systematically selection of participants 3. The incomplete answer was addressed, removing these answers   Unstatndaridized and Standard coffient Line 228 |
| Study size | 10 | Explain how the study size was arrived at | 7 | The sample size was determined by using a single population proportion formula. The proportion of faecal coliform (*E. coli*) contamination (P) (0.846) was chosen from a previous study conducted elsewhere  After considering a 95% of confidence interval (z=1.96) and 5% marginal error (d=0.05), and a 10% non-response rate, the final sample size was consolidated to be 220. Line 143 |

Continued on next page

| Quantitative variables | 11 | Explain how quantitative variables were handled in the analyses. If applicable, describe which groupings were chosen and why | 10 | Method of handling variables was reported. The criteria for selecting parameter and groups were detailed | |
| --- | --- | --- | --- | --- | --- |
| Statistical methods | 12 | (*a*) Describe all statistical methods, including those used to control for confounding | 11 | Descriptive analysis of data was conducted,  differences between groups were assessed using chi-squer or Fisher’s exact test or unpaired t-test or Mann-Whitney U test as appropriate Line 231  ANOVA, multiple linear regression  The level of significance was set at α = 5% and 95% confidence intervals (CI) were calculated line 127 -143 |  |
|  |  | (*b*) Describe any methods used to examine subgroups and interactions | 12 | This is described in the method section.  In this study multicolinearity was checked by using Pearson correlation coffient, the tolerance level and the variance inflation factors(VIF) line 245 |  |
|  |  | (*c*) Explain how missing data were addressed | 12 | Missing values were not inferred Line 252 |  |
|  |  | (*d*) *Cohort study*—If applicable, explain how loss to follow-up was addressed  *Case-control study*—If applicable, explain how matching of cases and controls was addressed  *Cross-sectional study*—If applicable, describe analytical methods taking account of sampling strategy |  |  |  |
|  |  | (*e*) Describe any sensitivity analyses |  | Non applicable Non applicable |  |
| Results | | | | |  |
| Participants | 13* | (a) Report numbers of individuals at each stage of study—eg numbers potentially eligible, examined for eligibility, confirmed eligible, included in the study, completing follow-up, and analysed | 12 | This is described at the beginning of result section  “A total of two hundred and twenty pairs of caregivers and babies from the three health institutions participated, with a response rate of 100%  -The contents of the baby bottles were categorized into four common food types: commercial powdered milk, cow's milk, cereal blend gruel, and fruit juice |  |
|  |  | (b) Give reasons for non-participation at each stage |  |  |  |
|  |  | (c) Consider use of a flow diagram |  | Use of a flow diagram was not deemed appropriate |  |
| Descriptive data | 14* | (a) Give characteristics of study participants (eg demographic, clinical, social) and information on exposures and potential confounders | 13-14 | Table 1 describes the participants and programs included in Line 264 to 299  ‘The mean ages of caregivers and babies were 30.09 ± 6.4 years and 30.09 ± 6.4 months, respectively  The monthly income of 25.5% of caregivers or mothers exceeded 5000 birrs  reasons for attending health institutions were routine vaccination in 32.7% of the participants  Most of the respondents were the biological mothers of the participants (76.4%) |  |
|  |  | (b) Indicate number of participants with missing data for each variable of interest | 13-18 | The total numbers of recorded data for each variable are stated in variable headline of each table |  |
|  |  | (c) *Cohort study*—Summarise follow-up time (eg, average and total amount) |  |  |  |
| Outcome data | 15* | *Cohort study*—Report numbers of outcome events or summary measures over time |  |  |  |
|  |  | *Case-control study—*Report numbers in each exposure category, or summary measures of exposure |  |  |  |
|  |  | *Cross-sectional study—*Report numbers of outcome events or summary measures | *15-18* | Frequency, percentage, mean standard deviation |  |
| Main results | 16 | (*a*) Give unadjusted estimates and, if applicable, confounder-adjusted estimates and their precision (eg, 95% confidence interval). Make clear which confounders were adjusted for and why they were included | 15 -18 | All results were reported in five tables and two figures  Mean , SD and 95%CI of TVC  Mean , SD and 95%CI of TVC  Proportion of detected food-borne pathogens  Corrected for food type, food hygiene practice and demographic characteristic or Unadjusted and adjusted estimates and their precision are reported |  |
|  |  | (*b*) Report category boundaries when continuous variables were categorized | 15-18 | Tabe 1 and table 2  Age was categorized  Bacterial count was also catagorized as   - TVC >5 log was an acceptable quality - TCC >2 log was an acceptable quality |  |
|  |  | (*c*) If relevant, consider translating estimates of relative risk into absolute risk for a meaningful time period |  |  |  |

Continued on next page

| Other analyses | 17 | Report other analyses done—eg analyses of subgroups and interactions, and sensitivity analyses | | | | |  |  | | | |
| --- | --- | --- | --- | --- | --- | --- | --- | --- | --- | --- | --- |
| Discussion | | | | | | | | |  |  |  |
| Key results | 18 | Summarise key results with reference to study objectives | | 18-23 | Key results are described at the beginning of discussion section (page 18), and later on by the mean of a paragraph displaying the main meaning and implications. The overall findings of this study revealed that 57.3 and 60.5% of the food samples had bacterial counts above the maximum standard values set for TVC and TCC, respectively, and were therefore deemed unacceptable in terms of microbial quality. In addition, approximately 8.63% (n = 19) of the samples contained foodborne pathogens and were therefore considered potentially hazardous for consumption. They also are summarized in the conclusion | | | |  |  |  |
| Limitations | 19 | Discuss limitations of the study, taking into account sources of potential bias or imprecision. Discuss both direction and magnitude of any potential bias | 23 | | | Description of limitations is done under limitation on page 23  “Institution based study Cross section not covered the whole food chain Limited surrogate of Hygiene indictors pathogens, Limited number and spectrum of food borne pathogens | | | | |  |
| Interpretation | 20 | Give a cautious overall interpretation of results considering objectives, limitations, multiplicity of analyses, results from similar studies, and other relevant evidence | | 24 | Relevant References were added where possible and discussed. Limitations were taken into account in the discussion.  The finding from this study showed the food safety risk associated with baby feeding bottle food at the time of consumption  The Hygiene standard of baby feeding bottle food preparation are also identified as unsatisfactory or sub-standard  Factors attributed for the bacterial load on baby feeding bottle food include  Lack of sterilisation of feeding bottle, and not washing hands during food preparation were the independent determinant of bacterial load  Type of food where cereal blend and fruit juice with the highest risk  However, ,the interpretation of the results was very cautious, given the cross-sectional nature of the study does not allow to infer causality | | | | |  |  |
| Generalizability | 21 | Discuss the generalizability (external validity) of the study results | | 25 | With all the stated limitations the study finding can be generalizable to a greater exent for similar settings, ie., in line with socioeconomic status.  We have used a large sample size, robust methods, and QA procedure during data collection and analysis to generalize its maximum level.  Moreover the study approach and the methods can also be duplicated in any further study to assess quality and safety  Overall, our results are highly generalizable for low-income countries in Africa, line 533 | | | | |  |  |
| Other information | |  | | | | | | | |  |  |
| Funding | 22 | Give the source of funding and the role of the funders for the present study and, if applicable, for the original study on which the present article is based | | 27 | No funds were received for this study | | | | |  |  |

*Give information separately for cases and controls in case-control studies and, if applicable, for exposed and unexposed groups in cohort and cross-sectional studies.

**Note:** An Explanation and Elaboration article discusses each checklist item and gives methodological background and published examples of transparent reporting. The STROBE checklist is best used in conjunction with this article (freely available on the Web sites of PLoS Medicine at http://www.plosmedicine.org/, Annals of Internal Medicine at http://www.annals.org/, and Epidemiology at http://www.epidem.com/). Information on the STROBE Initiative is available at www.strobe-statement.org.
